# Can spatial patterns along climatic gradients predict ecosystem responses to climate change? Experimenting with reaction-diffusion simulations

**DOI:** 10.1371/journal.pone.0174942

**Published:** 2017-04-10

**Authors:** Elena Roitberg, Maxim Shoshany

**Affiliations:** Faculty of Civil and Environmental Engineering, Technion—Israel Institute of Technology, Haifa, Israel; University of Oregon, UNITED STATES

## Abstract

Following a predicted decline in water resources in the Mediterranean Basin, we used reaction-diffusion equations to gain a better understanding of expected changes in properties of vegetation patterns that evolve along the rainfall transition between semi-arid and arid rainfall regions. Two types of scenarios were investigated: the first, a discrete scenario, where the potential consequences of climate change are represented by patterns evolving at discrete rainfall levels along a rainfall gradient. This scenario concerns space-for-time substitutions characteristic of the rainfall gradient hypothesis. The second, a continuous scenario, represents explicitly the effect of rainfall decline on patterns which evolved at different rainfall levels along the rainfall gradient prior to the climate change. The eccentricity of patterns that emerge through these two scenarios was found to decrease with decreasing rainfall, while their solidity increased. Due to their inverse modes of change, their ratio was found to be a highly sensitive indicator for pattern response to rainfall decline. An eccentricity ratio versus rainfall (ER:R) line was generalized from the results of the discrete experiment, where ERs above this line represent developed (recovered) patterns and ERs below this line represent degraded patterns. For the rainfall range of 1.2 to 0.8 mm/day, the continuous rainfall decline experiment with ERs that lie above the ER:R line, yielded patterns less affected by rainfall decline than would be expected according to the discrete representation of ecosystems’ response. Thus, for this range, space-for-time substitution represents an overestimation of the consequences of the expected rainfall decline. For rainfall levels below 0.8 mm/day, eccentricity ratios from the discrete and continuous experiments practically converge to the same trend of pattern change along the ER:R line. Thus, the rainfall gradient hypothesis may be valid for regions characterized by this important rainfall range, which typically include desert fringe ecosystems.

## Introduction

The Mediterranean Basin is recognized in a distinctive number of studies as a hot-spot of climate change [1–3]. Mariotti et al. ([3]) predicted a 10–30% decrease in water resources in the Eastern Mediterranean by the end of this century, due to the combined effect of decreasing rainfall and increasing atmospheric temperatures.

Studying the potential consequences of such a decrease poses a fundamental challenge due to the high ecological, hydrological, and geomorphological diversity of Mediterranean regions [4]. Research in this field concentrates on three approaches: manipulation experiments, simulation models, and space-for-time representations [5]. Despite the limitations of these approaches (e.g., [6–7]), all three were used to improve our understanding of future impacts of climate change in a distinctive number of studies (e.g., [8–9]). Miranda et al. ([4]), who based their research on earlier studies, suggested that for decreasing rainfall levels of between 30%, and 80% in semi-arid shrub steppe, aboveground productivity is linearly correlated to annual precipitation. Such findings support the extensive use of precipitation gradients for predicting responses of ecosystems to climate changes (e.g., [10]). On the other hand, a more recent study [5] claimed that “inferences based on space-for-time substitution overestimate the magnitude of responses to contemporary climate warming, because spatial gradients reflect long-term processes”. Further assessment of rainfall gradients as space-for-time transformations that facilitate the prediction of ecosystem responses to climate change is therefore undoubtedly required. Within such an assessment, changes in patch pattern properties along these processes should be examined; as such changes may serve as early indicators of climate change [11, 12]. Historical information sources such as aerial photographs and manipulation experiments are of limited spatial extent and temporal range, and their value for such assessments is, therefore, limited as well.

Geosimulations in general (see extensive review in [12, 13]), and reaction-diffusion simulations in particular, may facilitate the analysis of patch pattern responses to changes in water availability (e.g., [14]). This approach was implemented by researchers studying the formation and change of patterns along rainfall gradients under desertification (e.g., [15–20]); however, relatively few studies have examined the change in pattern characteristics due to decreasing or increasing water availability. Indicators mentioned within this context include, for example, truncation of power-law relationships (e.g., [21–22]), changes in the spatial frequencies of stripes (e.g. [23–25]), and migration of stripes [19, 26–27]. Variations in water availability (rainfall) involve shifts between spotty and striped patterns through myriad forms of different spatial complexity (e.g., [17, 28–33]). While spotty patterns are characterized by high solidity (similarity of a patch to its convex hull) and low elongation, striped patterns are typically of low solidity and high elongation. We thus hypothesize that metrics of eccentricity and solidity are sensitive to pattern changes associated with decreasing rainfall. The sensitivity of these pattern properties to rainfall changes are expected to be instrumental in identifying areas affected by decrease in water resources, and in assessing the extent to which current rainfall pattern relationships represent future pattern responses to rainfall decline.

With the exception of studies that examined the spatial properties of gaps between vegetation patches, most of the existing work that used landscape metrics represents the form of vegetation patches while disregarding the bare soil pattern configuration. Shoshany ([34]) and Shoshany and Kelman ([35]) stressed the importance of mutually analyzing the pattern properties of both vegetation and soil patch configurations so as to gain a better understanding of habitat transformations. Here, by implementing reaction-diffusion equations (RDEs), we will examine the eccentricity and solidity of shrub and soil patch patterns, in response to decreasing rainfall levels, as potential indicators of rainfall decline. These simulations will also serve in the assessment of the rainfall gradient hypothesis of space-for-time transformations, by comparing discrete and continuous modes of pattern responses to rainfall change. The discrete mode will be realized by simulating pattern evolution at discrete rainfall levels; responses to climate change will be informed only implicitly, through differences in the pattern properties. The continuous mode of change will be realized by the change in the patterns that emerged in the discrete mode following a simulated decrease in rainfall, thus representing rainfall change in an explicit manner. To the best of our knowledge, RDEs had not been utilized yet in assessing the space-for-time hypothesis.

### Reaction-diffusion patch pattern dynamics

Since the pioneering work of Turing ([36]), reaction-diffusion equations (RDEs) have been implemented in many diverse fields of pattern-forming processes. Lefever and Lejeune ([37]) were among the first to apply RDEs to better understand the changes in water-limited ecosystem patterns and to explain the formation of spots, labyrinths, rings, and banded stripes. Our work implements the model first developed by Hille Ris Lambers et al. ([38]), which is still in wide use [14, 39–41]. In this model, vegetation patterning is linked to the mechanism of positive feedback between plant density and water infiltration [17, 26, 29, 42–49]. The model is typically composed of three partial differential equations that describe the dynamics of three state variables: plant density, soil water, and surface water, as a function of plant growth, plant loss, and plant dispersal, which depend on rainfall intensity, runoff and overland flow, soil infiltration, evaporation, and plant water uptake (see details in S1 Appendix as adapted from Rietkerk, 2002):

[change in plant density]=[plant growth]-[plant loss]±[plant dispersal] (1A)

[change in soil water]=[infiltration rate]-[plant water uptake]-[evaporation and drainage]±[water movement] (1B)

[change in surface water]=[rainfall rate]-[infiltration rate]±[overland flow] (1C)

### Pattern shape analysis

Substantial work has been done in the study of landscape ecology concerning the use of landscape metrics for studying affinities between forms of spatial processes, their ecological mechanisms, and their driving forces [50–51]; however, research on the use of such metrics in the study of climate change is limited. We suggest characterizing the spatial forms of the vegetation or bare soil patches with solidity and eccentricity parameters, where solidity (S) is the ratio between the area of an object and its convex area, and eccentricity (E) is the ratio between the major and minor axes of the best fitting ellipse (detailed information is given in S2 Appendix). During the transformation of spotty patterns into banded patterns and then into convoluted labyrinths, patches lose their solidity and become more elongated and connected. Our hypothesis is that the use of eccentricity and solidity metrics may improve morphometric sensitivity to pattern changes associated with changes in water availability. Following the assessment of these metrics and their inverse mode of change, we suggest a new metric that represents the ratio between these two original metrics which facilitates improved sensitivity to pattern changes.

## Materials and methods

### Experimental design

**Fig 1:**
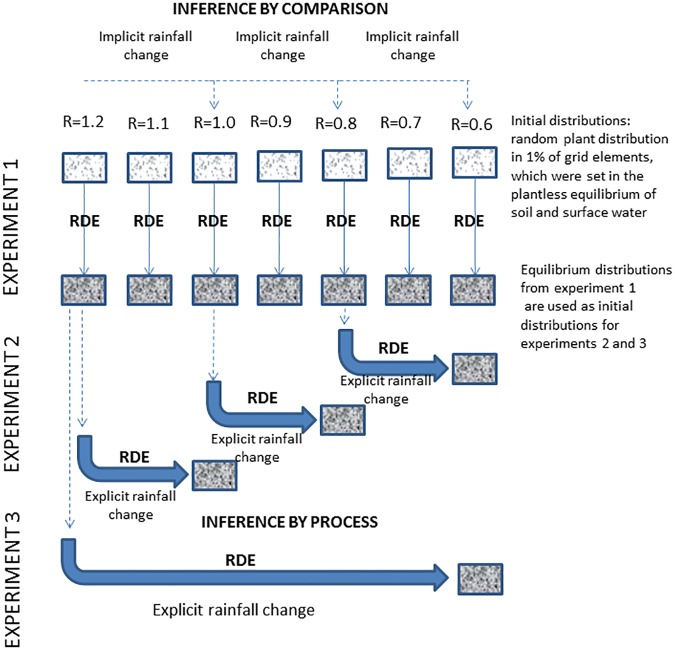
Schematic presentation of the research methodology.

Three sets of simulated scenarios (experiments) were developed to compare patterns that reach equilibrium at discrete rainfall levels, which implicitly represent rainfall change, with patterns that explicitly represent the continuous response mode of the ecosystem (Fig 1) to rainfall decline. Sequences of patch pattern maps (image chains), 400m x 400m, were produced for each scenario by running RDEs.

#### Experiment 1

Experiment 1 represented the discrete rainfall gradient scenario (DGS) in which RDE simulations were carried out separately for seven discrete rainfall levels (between 1.2 and 0.6 mm/day). Initial spatial plant distribution for each run consisted of random plant distribution in 1% of grid elements, which were set in the plantless equilibrium of soil and surface water. Simulations were stopped when the change in all observed parameters was smaller than the prescribed threshold, i.e. the pattern evolution had reached equilibrium. The continuous mode of rainfall change was assessed by applying rainfall decrease on the equilibrium patterns reached at the discrete rainfall levels. The decrease in rainfall simulated here follows the rainfall change trends reported by Philandras et al. ([52]) and Mariotti et al. ([3]) for the Mediterranean region.

#### Experiment 2 and experiment 3

The second and third experiments simulated two types of continuous rainfall change. In Experiment 2, a moderate continuous rainfall reduction scenario was applied on equilibrium patterns that represent Mediterranean semi-arid (1.2 mm/day), hot semi-arid (1 mm/day), and desert fringe (0.8 mm/day) ecoregions. Plant distribution and soil and surface water of patterns that reached equilibrium at these rainfall levels in Experiment 1 were subjected to iterative changes in rainfall. In each iteration, the annual rainfall level was decreased by 36.5 mm (0.1 mm/day). Simulations at this level continued until the pattern reached equilibrium and only then did we progress to the next iteration with a lower rainfall level. Earlier assessments of such simulations indicated that equilibrium is reached after 5 to 8 simulated years. Dividing the reduction of 36.5 mm by the number of simulated years, yields an annual change of 4.5 to 7.3 mm. These rates are comparable to the 2–6 mm per year rates reported by Philandras et al. ([52]) for the Mediterranean region.

Experiment 3 simulated the desertification of Mediterranean ecoregions by gradually decreasing rainfall from Mediterranean semi-arid levels (1.2 mm/day) to desert levels (0.6 mm/day). Initial conditions (plant distribution, soil and surface water) for this scenario were represented by the pattern that reached equilibrium at the 1.2 mm/day rainfall level in Experiment 1. Similar to Experiment 2, rainfall was decreased in each iteration by 0.1 mm/day and the RDE simulation continued until the process reached equilibrium for this rainfall level, before progressing to the next rainfall reduction level (next iteration). The iterations were stopped when rainfall reached 0.6 mm/day.

## Results

### Pattern characteristics for three rainfall intervals

**Fig 2:**
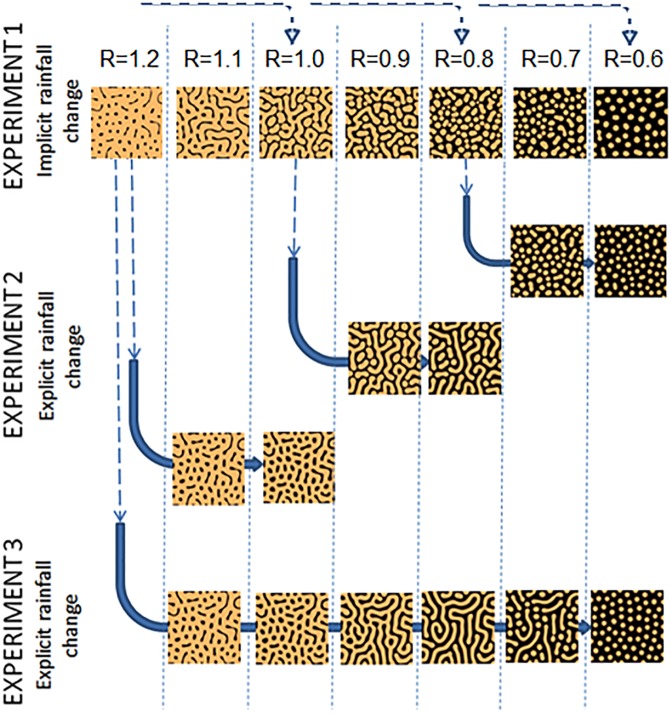
Patch pattern maps at equilibrium, obtained from the RDE model at various rainfalls in three experiments.

**Fig 3:**
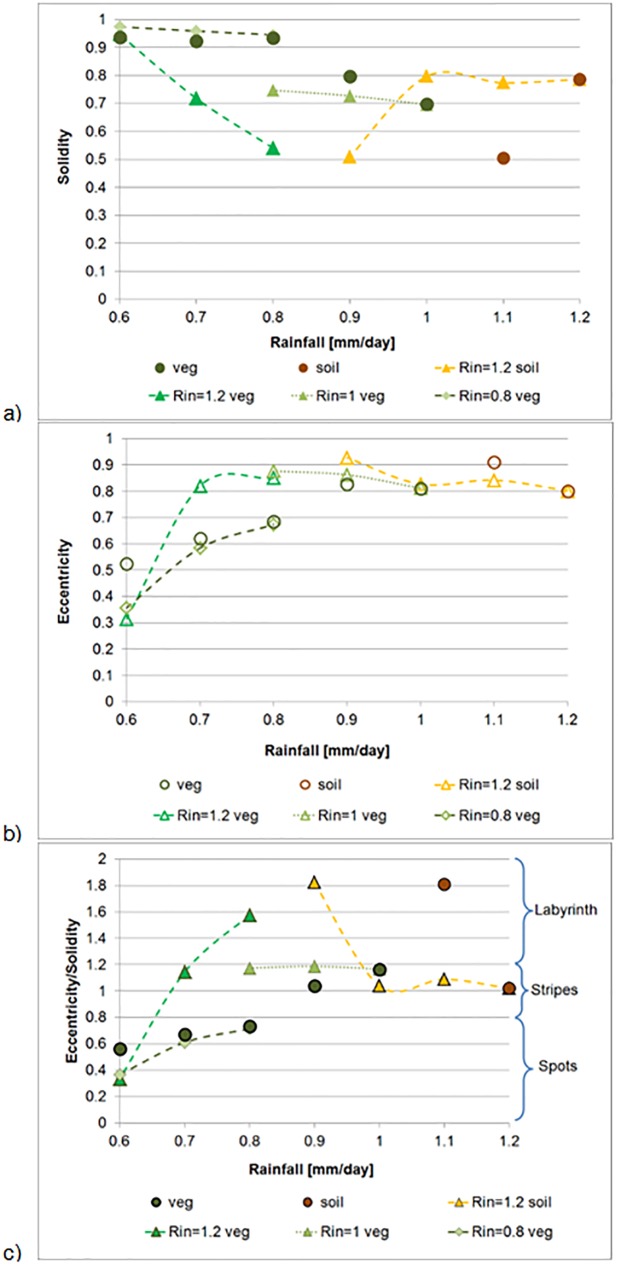
Vegetation and soil patch properties as a function of rainfall. **A) Solidity; B) Eccentricity, and C) Eccentricity and solidity ratio.** Individual point symbols: results of Experiment 1. Dashed lines: the evolution of pattern properties in Experiments 2 and 3. Rin: the initial rainfall for Experiments 2 and 3.

Pattern sequences produced in the discrete experiment follow the same mode of change as reported in earlier studies that used RDEs. In general terms, these patterns may be divided into the following categories, each of which characterizes a different rainfall range (Figs 2, 3A and 3B):

- Rainfall under 0.8 mm/day: Patterns in this rainfall range are primarily spotted, with small and medium size vegetation patches scattered over the bare ground. Solidity is maintained high, while eccentricity decreases with the decreasing rainfall.
- Rainfall over 1 mm/day: This range is characterized by dominant vegetation cover and pattern properties that are best represented by the characteristics of the soil patch distribution. Higher levels of rainfall are incorporated by a lower proportion of bare soil with a highly connected vegetation network at 1 mm/day rainfall and a spotty pattern at 1.2 mm/day rainfall. Differences in eccentricity and solidity represent well the differences between these patterns, whereby the stripped pattern is characterized by high eccentricity and low solidity, and the spotty pattern by high solidity and slightly lower eccentricity.
- Intermediate rainfall levels between 1 and 0.8 mm/day: This rainfall range is characterized by highly connected soil and vegetation networks. At the 0.8 mm/day rainfall level, vegetation stripes are more often truncated, with higher solidity and lower eccentricity levels, while at the 1 mm/day level, the pattern of vegetation stripes has lower solidity and higher eccentricity.

#### Trends of eccentricity ratio change

In general, eccentricity and solidity vary inversely in response to changing rainfall levels (Fig 3A and 3B). Thus, the combined eccentricity ratio (ER) index decreases linearly with the decrease in rainfall, for rainfall levels equal or lower than 1 mm/day (Fig 3C). The high slope of the ER vs R line indicates the high sensitivity of the new metric to rainfall change.

**Fig 4:**
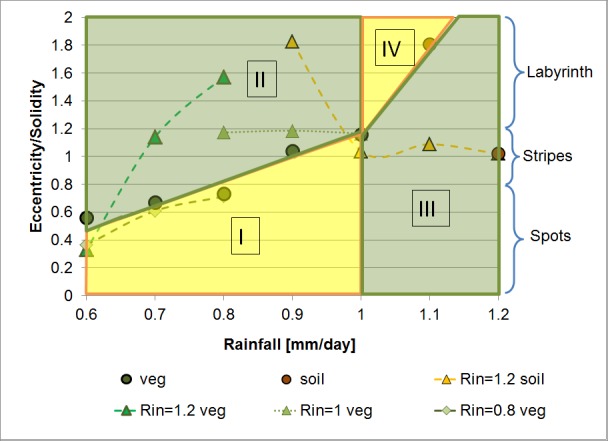
The division of the eccentricity ratio vs rainfall into four regions: Green regions are favorable for vegetation growth and yellow for bare soil distribution. This generalized scheme is instrumental in the interpretation of results from the two continuous experiments:

Fig 4 presents the division of the eccentricity ratio versus rainfall space into four regions based on the intersection between the eccentricity ratio line and the line separating vegetation dominance from soil dominance, at a rainfall level of 1 mm/day:

- Region I is the region below the ER line and to the left of the 1 mm/day rainfall line. This region represents recovering patterns prior to equilibrium or degraded patterns due to human disturbance or a decrease in habitat resources.
- Region II lies above the ER line and to the left of the 1 mm/day rainfall line. This region represents "developed" vegetation patterns of larger patches and/or higher connectivity than patterns characteristic of corresponding rainfall levels along the ER:R line.
- Region III is the region below the ER line and to the right of the 1 mm/day rainfall line, and it represents favorable vegetation conditions due to vegetation dominance and high fragmentation of soil patches.
- Region IV lies above the ER line and to the right of the 1 mm/day rainfall line. This region is characterized by higher connectivity of soil patches than in patterns that characterize Region III at corresponding rainfall levels.

Moderate continuous rainfall decrease scenario (-0.2 mm/day):

- 1.2 to 1 mm/day: This sequence is mapped in the vegetation domination region (III) with soil patches remaining primarily spotty. Throughout the continuous rainfall decrease experiment, the soil pattern remained highly fragmented, with an almost constant eccentricity ratio (~1).
- 1 to 0.8 mm/day: The labyrinth-like vegetation-soil patterns evolved in the discrete rainfall experiment at 1 mm/day rainfall and were maintained throughout this rainfall interval. ERs are within Region II and remain almost constant (~1.2).
- 0.8 to 0.6 mm/day: Vegetation patterns are highly spotty. ERs decrease following the ER:R line as defined for the discrete experiment results.

Extended continuous rainfall decrease scenario (from 1.2 to 0.6 mm/day):

The fragmented soil pattern obtained at 1 mm/day in the moderate continuous rainfall decrease scenario was transformed into a labyrinth-like pattern at a rainfall level of 0.9 and was maintained also at 0.8 mm/day. Further decrease in rainfall resulted in the progressive dissection of the long vegetation patches into short stripes at 0.7 mm/day and into a spotty pattern at 0.6 mm/day. The ERs of patterns that emerged in this experiment lie within Region II at levels significantly higher than the ER:R line, expressing the highly connected network of vegetation patches maintained almost throughout the entire range of rainfall decrease. ER levels in this experiment were the highest (~1.8), reaching well above the maximum level obtained in Experiments 1 and 2 for vegetation (1.2). The rate of decrease in ER is also very high at decreasing rainfall levels below 0.8 mm/day.

## Discussion

Differences between the continuous and discrete rainfall decrease scenarios are most distinctive along the rainfall gradient between 1.2 and 0.8 mm/day. While there was a consistent and significant change in the ERs obtained at the discrete rainfall levels, only minor or negligible decreases in ERs were exhibited in the continuous experiments:

- Soil patch fragmentation remained almost unchanged during the decrease in rainfall from 1.2 mm/day to 1 mm/day, yielding a pattern of higher vegetation connectivity at 1 mm/day was than obtained at this rainfall level in the discrete experiment.
- Labyrinth-like vegetation patch continuity was maintained despite a reduction in rainfall from 1 mm/day to 0.8 mm/day.

These results suggest that vegetation patches facing a decrease in rainfall sustain the change in water availability due to their improved plant-soil conditions (e.g., organic matter content and deep root systems), which evolved prior to the decrease in water levels. In other words, there is a kind of "inheritance" of previous growth conditions that moderates the response of vegetation to changes in rainfall: this is the “ecosystem resistance against catastrophic shifts” suggested by Rietkerk et al. [19]. Our findings for this rainfall range thus support the claim made by Elemendorf et al. ([5]) that “inferences based on space-for-time substitution overestimate the magnitude of responses to contemporary climate warming”.

Patterns at 0.8 mm/day rainfall differed significantly between the experiments: the pattern obtained in the discrete experiment was patchy, while those obtained in the two continuous experiments were labyrinth-like. With a 0.1 mm/day decrease in rainfall, however, the pattern becomes spotty in all three experiments and the trends of change converge. The information from the discrete patterns for the range between 0.8 and 0.6 mm/day may serve as close approximation for pattern properties characteristic of continuous rainfall decrease. The space-for-time approximations along rainfall gradients seem, therefore, to be valid for this range, which represents rainfall levels at the desert fringe.

## Summary and conclusions

Reaction-diffusion equations (RDE) were used with the aim of gaining a better understanding of the expected changes in the properties of patch patterns that evolve along a rainfall transition between semi-arid and arid rainfall regions following a predicted decline in water resources in the Mediterranean Basin. The characteristic equilibrium patterns that emerge as a result of the three rainfall regimes are: spotty vegetation patterns at rainfall levels lower than 0.8 mm/day, transition between spotty vegetation and spotty soil patterns through striped and labyrinth-like patterns for rainfall levels between 0.8 and 1 mm/day, and high vegetation cover together with spotty and striped soil patterns at rainfall levels higher than 1 mm/day. Pattern characteristics of eccentricity and solidity responded inversely to the decline in rainfall, and the ratio between the two parameters is suggested to be an efficient morphometric indicator. The eccentricity ratio versus rainfall line (ER:R line), together with a line that distinguishes between soil dominance and vegetation dominance at the 1 mm/day rainfall level allows to differentiate between four typologies of developed and degraded patterns. For the range between 1.2 and 0.8 mm/day rainfall, the continuous rainfall decline yielded patterns with higher vegetation connectivity than patterns that evolved at corresponding discrete rainfall levels, as inferred by ERs above the ER:R line. We hypothesize that this result represents "pattern inheritance" of plant-soil relationships that evolved prior to the decline in rainfall levels. Thus, for this range, space-for-time substitution represents an overestimation of the consequences of the expected rainfall decline (e.g., [5]). This result is also in line with suggestions made by Dunkerley ([53]) regarding the robustness of banded vegetation patterns to both grazing pressures and rainfall change. For rainfall levels lower than 0.8 mm/day, the eccentricity ratios from the discrete and continuous experiments converge practically to the same trend of pattern change along the ER:R line. Thus, the rainfall gradient hypothesis may be valid for regions within this important rainfall range, which typically includes desert fringe ecosystems.

This research follows the line of assessing primarily equilibrium patterns based on simulations that represent changes in water availability. Human disturbances significantly increase the complexity of relationships between patterns and habitat conditions. As Tietjen and Jeltsch ([12]) suggest, these effects require looking into as well. Several recent studies that included simulations of recovery following disturbance (e.g., [4, 40]) found that there is an inheritance aspect also to patterns that are recovering from disturbance. Yet it is well understood that human disturbances add almost infinitesimal forms of pattern variation and real difficulties in distinguishing climate-induced changes from human-induced changes. With the growing availability of remote sensing data at high spatiotemporal resolutions, it is possible to broaden these assessments into real pattern sequences in which environmental information may help minimize uncertainty regarding the causes of pattern change. These avenues of research will be addressed in future phases of our research in this field.

## Supporting information

S1 Appendix Reaction-diffusion model equations. Table A in S1 Appendix. Parameters for the RDE model as adapted from Rietkerk (2002)
(DOCX)

S2 Appendix Calculation of eccentricity and elongation indices. Fig A in S2 Appendix. Characteristic patterns and their shape properties. Left side: (a, b, c) landscape patterns obtained from simulations. Right side: calculated shape parameters for the patterns on left. Table A in S2 Appendix. Characteristic parameters for different geometric shapes.
(DOCX)

S1 File Patch pattern maps. This file contains patch pattern maps that were obtained from the implementation of the RDE model along three experiments. These maps represent initial stages and final states when patterns reach equilibrium
(ZIP)

S2 File The analyzed Excel data. (XLS)
