# Supplementary material for: Can spatial patterns along climatic gradients predict ecosystem responses to climate change? Experimenting with reaction-diffusion simulations
Source: PLoS One. 2017 Apr 10;12(4):e0174942. doi: 10.1371/journal.pone.0174942 (PMC5386256; doi:10.1371/journal.pone.0174942)
Supplement: S1 Appendix — Table A in S1 Appendix. Parameters for the RDE model as adapted from Rietkerk (2002) (DOCX) [file pone.0174942.s001.docx]

**S1 Appendix: Reaction-Diffusion Model Equations**

| $\frac{\partial P\left( \vec{x},t \right)}{\partial t}=\left[ \begin{aligned} \text{plant growth}\text{ } \\ \text{at} \vec{x} \text{at} t \end{aligned} \right]-\left[ \begin{matrix} plant loss \\ \text{at} \vec{x} \text{at} t \end{matrix} \right]\pm\left[ plant dispersal \right]=cg_{max}\frac{W}{W+k_{1}}P-dP+D_{P}\Delta P$ | $(1a)$ |
| --- | --- |
| $\frac{\partial W\left( \vec{x},t \right)}{\partial t}=\left[ \begin{matrix} infiltration rate \\ \text{at} \vec{x} \text{at} t \end{matrix} \right]-\left[ \begin{matrix} plant water uptake \\ \text{at} \vec{x} \text{at} t \end{matrix}\text{ } \right]-\left[ \begin{matrix} evaporation and drainage \\ \text{at} \vec{x} \text{at} t \end{matrix} \right]$  $\pm\left[ water movement \right] =\alpha O\frac{P+k_{2}W_{0}}{P+k_{2}}-g_{max}\frac{W}{W+k_{1}}P-r_{w}W+D_{W}\Delta W$ | $(1b)$ |
| $\frac{\partial O\left( \vec{x},t \right)}{\partial t}=\left[ \begin{matrix} rainfall rate \\ \text{at} \vec{x} \text{at} t \end{matrix}\text{ } \right]-\left[ \begin{matrix} infiltration rate \\ \text{at} \vec{x} \text{at} t \end{matrix} \right]\pm\left[ overland flow \right]=R-\alpha O\frac{P+k_{2}W_{0}}{P+k_{2}}+D_{O}\Delta O$ | $(1c)$ |

**Table A. Parameters for the RDE model as adapted from Rietkerk (2002).**

| Parameter | Description | |
| --- | --- | --- |
| $D_{P}=0.1m^{2}/day$ | Plant dispersal | |
| $D_{W}=0.1m^{2}/day$ | | Diffusion coefficient for soil water |
| $D_{O}=100m^{2}/day$ | | Diffusion coefficient for surface water |
| *O* [*mm* ] | | Surface water |
| *P* [$g/m^{2}$] | | Plant density |
| $R [mm/day ]$ | | Rainfall (ranged between 0 to 3) |
| *W* [*mm*] | | Soil water |
| $W_{0}=0.2$ | | Water infiltration rate in absence of plants |
| $c=10g/(mm\cdot m)$ | | Conversion of water uptake by plants to plant growth |
| $d=0.25\frac{1}{day}$ | | Specific loss of plant density due to mortality |
| $g_{max}=0.05mm/(g\cdot m^2\cdot day)$ | | Maximum specific water uptake |
| $k_{1}=5mm$ | | Half-saturation constant of specific plant growth and water uptake |
| $k_{2}=5 g/m^{2}$ | | Saturation constant of water infiltration |
| $r_{w}=0.2\frac{1}{day}$ | | Specific soil water loss due to evaporation and drainage |
| $\alpha=0.2\frac{1}{day}$ | | Maximum infiltration rate |
