# Supplementary material for: Can spatial patterns along climatic gradients predict ecosystem responses to climate change? Experimenting with reaction-diffusion simulations
Source: PLoS One. 2017 Apr 10;12(4):e0174942. doi: 10.1371/journal.pone.0174942 (PMC5386256; doi:10.1371/journal.pone.0174942)
Supplement: S2 Appendix — Fig A in S2 Appendix. Characteristic patterns and their shape properties. Left side: (a, b, c) landscape patterns obtained from simulations. Right side: calculated shape parameters for the patterns on left. Table A in S2 Appendix. Characteristic parameters for different geometric shapes. (DOCX) [file pone.0174942.s002.docx]

**S2 Appendix: Calculation of Eccentricity and Elongation Indices**

- **Solidity** **(S)** represents the convexity ratio, which is the ratio of the area of an object to its convex area, i.e., the smallest convex region that contains the original region (the convex hull is what will be obtained if a rubber band is wrapped around the region, for example, the convex object of
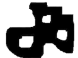
 is
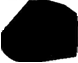
).

| $Solidity=\frac{Region Area}{ConvexArea}$ | $(2)$ |
| --- | --- |
| Solidity ranges between 0 and 1. |  |

- **Eccentricity (E)** is the ratio between the major and minor axes of the best fitting ellipse, which has the same second-moments as the region. The ratio of the length of the longest chord of the shape to the longest chord perpendicular to it. Eccentricity ranges between 0 and 1 and it characterizes the degree of elongation of the object:

| $Eccentricity=\frac{2\cdot\sqrt{\left( 0.5A_{max} \right)^{2}-\left( 0.5A_{min} \right)^{2}}}{A_{max}}$ | $(3)$ |
| --- | --- |

where $A_{max}$ and $A_{min}$ are the major and minor axes of the best fitting ellipse, respectively, and are calculated as follows:

| $A_{max}=2\cdot\sqrt{2}\sqrt{U_{x}+U_{y}+C}$ | $(4a)$ |
| --- | --- |
| $A_{min}=2\cdot\sqrt{2}\sqrt{U_{x}+U_{y}-C}$ | $(4b)$ |

where $U_{x}$ and $U_{y}$are auxiliary magnitudes as defined below, *N* is the number of pixels related to the object, and $p\left( x,y \right)$represent the pixels belonging to object Ω.

The coordinates of the center of mass are calculated as:

| $x_{c}=\frac{1}{N}\sum_{p\left( x,y \right)\in\Omega} x$ | $\left( 5a \right)$ |
| --- | --- |
| $y_{c}=\frac{1}{N}\sum_{p(x,y)\in\Omega} y$ | $(5b)$ |
| $U_{x}=\frac{1}{12}+\frac{1}{N}\sum_{p(x,y)\in\Omega} {(x-x_{c})}^{2};$ | $(6a)$ |
| $U_{y}=\frac{1}{12}+\frac{1}{N}\sum_{p(x,y)\in\Omega} {(y-y_{c})}^{2}$ | $(6b)$ |
| $U_{xy}=\frac{1}{12}+\frac{1}{N}\sum_{p(x,y)\in\Omega} (x-x_{c})(y-y_{c})$ | $(6c)$ |
| $C=\sqrt{{(U_{x}-U_{y})}^{2}+4\cdot{U_{xy}}^{2}}$ | $(7)$ |

- **Eccentricity Ratio (ER)** is a metric which increases sensitivity to elongation change while solidity decreases:

| $ER=\frac{Eccentricity}{Solidity}$ | $(8)$ |
| --- | --- |

Table A presents solidity, eccentricity and eccentricity ratio values obtained for simple geometric shapes.

**Table A. Characteristic parameters for different geometric shapes.**

| Object | | Solidity | Eccentricity | Ratio |
| --- | --- | --- | --- | --- |
| Circle | 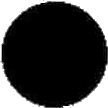 | 1 | 0 | 0 |
| Ellipse | 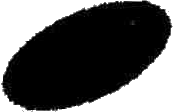 | 1 | 0.88 | 0.88 |
| Rectangle with aspect ratio 1:3 | 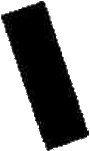 | 0.95 | 0.95 | 1 |
| Rectangle with aspect ratio 1:12 | 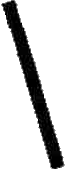 | 0.88 | 1 | 1.13 |
| S-shaped object | 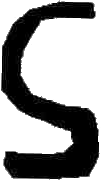 | 0.46 | 0.87 | 1.90 |

The above equations parameterize the form of an individual patch (single object). When considering multi-object regions, pattern properties (PP) are defined according to the following weighted-area sum for each metric parameter (solidity, eccentricity, and eccentricity ratio) for each image patch:

| $PP=\frac{\sum_{i=1}^{K} \mathrm{parameter}_{i}\cdot{Patch Area}_{i}}{\sum_{i=1}^{K} {Patch Area}_{i}}$ | $(9)$ |
| --- | --- |

Preliminary assessment of the three shape metrics was obtained by applying them to characteristic patterns (Fig A).


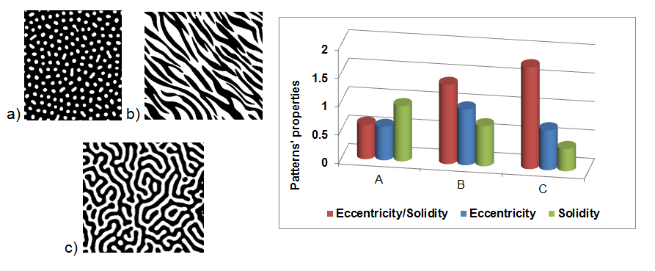


**Fig A. Characteristic patterns and their shape properties.** Left side: (a, b, c) landscape patterns obtained from simulations. Right side: calculated shape parameters for the patterns on left.

Parameterization of the vegetation and soil pattern configuration for each of the pattern maps produced by the RDEs during the experiments was performed according to the following procedure:

1. Delineation of individual vegetation and soil patches (e.g., Gonzalez et al. 2008).
2. Calculation of patch area.
3. Parameterization of solidity, eccentricity, and eccentricity ratio using Equations 2, 3 and 8 for each soil and vegetation patch (Appendix S2).
4. Determination of patch pattern properties using Equation 9 (Appendix S2).

The final products are sequences of the three pattern properties obtained for each of the three scenarios.

## Reference

Gonzalez RC, Woods RE. Digital Image Processing 3rd ed. 2008.
