# Supplementary figures and images for: Can spatial patterns along climatic gradients predict ecosystem responses to climate change? Experimenting with reaction-diffusion simulations
Source: PLoS One. 2017 Apr 10;12(4):e0174942. doi: 10.1371/journal.pone.0174942 (PMC5386256; doi:10.1371/journal.pone.0174942)

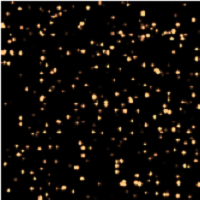

Supplement: S1 File — This file contains patch pattern maps that were obtained from the implementation of the RDE model along three experiments. These maps represent initial stages and final states when patterns reach equilibrium (ZIP) [file pone.0174942.s003.zip › S1 file_patch pattern maps/Patch pattern maps/experiment1/after 30days/R=0.6_30days.png]

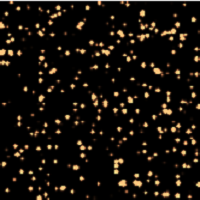

Supplement: S1 File — This file contains patch pattern maps that were obtained from the implementation of the RDE model along three experiments. These maps represent initial stages and final states when patterns reach equilibrium (ZIP) [file pone.0174942.s003.zip › S1 file_patch pattern maps/Patch pattern maps/experiment1/after 30days/R=0.7_30days.png]

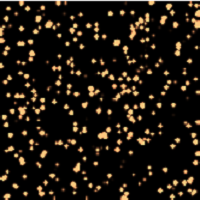

Supplement: S1 File — This file contains patch pattern maps that were obtained from the implementation of the RDE model along three experiments. These maps represent initial stages and final states when patterns reach equilibrium (ZIP) [file pone.0174942.s003.zip › S1 file_patch pattern maps/Patch pattern maps/experiment1/after 30days/R=0.8_30days.png]

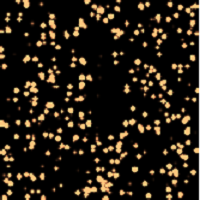

Supplement: S1 File — This file contains patch pattern maps that were obtained from the implementation of the RDE model along three experiments. These maps represent initial stages and final states when patterns reach equilibrium (ZIP) [file pone.0174942.s003.zip › S1 file_patch pattern maps/Patch pattern maps/experiment1/after 30days/R=0.9_30days.png]

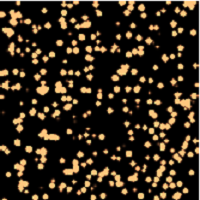

Supplement: S1 File — This file contains patch pattern maps that were obtained from the implementation of the RDE model along three experiments. These maps represent initial stages and final states when patterns reach equilibrium (ZIP) [file pone.0174942.s003.zip › S1 file_patch pattern maps/Patch pattern maps/experiment1/after 30days/R=1.1_30days.png]

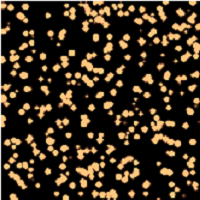

Supplement: S1 File — This file contains patch pattern maps that were obtained from the implementation of the RDE model along three experiments. These maps represent initial stages and final states when patterns reach equilibrium (ZIP) [file pone.0174942.s003.zip › S1 file_patch pattern maps/Patch pattern maps/experiment1/after 30days/R=1.2_30days.png]

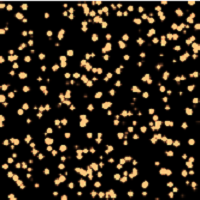

Supplement: S1 File — This file contains patch pattern maps that were obtained from the implementation of the RDE model along three experiments. These maps represent initial stages and final states when patterns reach equilibrium (ZIP) [file pone.0174942.s003.zip › S1 file_patch pattern maps/Patch pattern maps/experiment1/after 30days/R=1_30days.png]

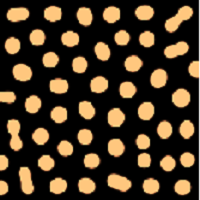

Supplement: S1 File — This file contains patch pattern maps that were obtained from the implementation of the RDE model along three experiments. These maps represent initial stages and final states when patterns reach equilibrium (ZIP) [file pone.0174942.s003.zip › S1 file_patch pattern maps/Patch pattern maps/experiment1/equilibrium/R=0.6_eq.png]

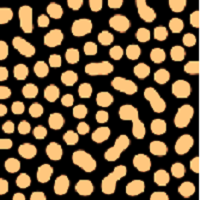

Supplement: S1 File — This file contains patch pattern maps that were obtained from the implementation of the RDE model along three experiments. These maps represent initial stages and final states when patterns reach equilibrium (ZIP) [file pone.0174942.s003.zip › S1 file_patch pattern maps/Patch pattern maps/experiment1/equilibrium/R=0.7_eq.png]

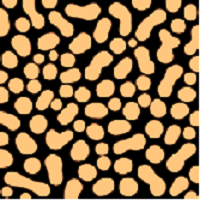

Supplement: S1 File — This file contains patch pattern maps that were obtained from the implementation of the RDE model along three experiments. These maps represent initial stages and final states when patterns reach equilibrium (ZIP) [file pone.0174942.s003.zip › S1 file_patch pattern maps/Patch pattern maps/experiment1/equilibrium/R=0.8_eq.png]

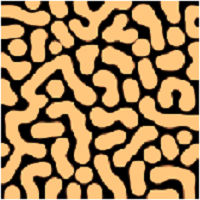

Supplement: S1 File — This file contains patch pattern maps that were obtained from the implementation of the RDE model along three experiments. These maps represent initial stages and final states when patterns reach equilibrium (ZIP) [file pone.0174942.s003.zip › S1 file_patch pattern maps/Patch pattern maps/experiment1/equilibrium/R=0.9_eq.png]

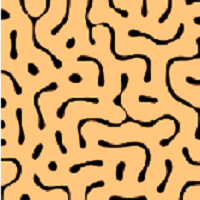

Supplement: S1 File — This file contains patch pattern maps that were obtained from the implementation of the RDE model along three experiments. These maps represent initial stages and final states when patterns reach equilibrium (ZIP) [file pone.0174942.s003.zip › S1 file_patch pattern maps/Patch pattern maps/experiment1/equilibrium/R=1.1_eq.png]

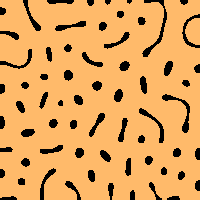

Supplement: S1 File — This file contains patch pattern maps that were obtained from the implementation of the RDE model along three experiments. These maps represent initial stages and final states when patterns reach equilibrium (ZIP) [file pone.0174942.s003.zip › S1 file_patch pattern maps/Patch pattern maps/experiment1/equilibrium/R=1.2_eq.png]

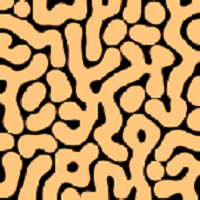

Supplement: S1 File — This file contains patch pattern maps that were obtained from the implementation of the RDE model along three experiments. These maps represent initial stages and final states when patterns reach equilibrium (ZIP) [file pone.0174942.s003.zip › S1 file_patch pattern maps/Patch pattern maps/experiment1/equilibrium/R=1_eq.png]

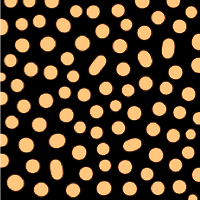

Supplement: S1 File — This file contains patch pattern maps that were obtained from the implementation of the RDE model along three experiments. These maps represent initial stages and final states when patterns reach equilibrium (ZIP) [file pone.0174942.s003.zip › S1 file_patch pattern maps/Patch pattern maps/experiment2/Rin=0.8_R=0.6eq.png]

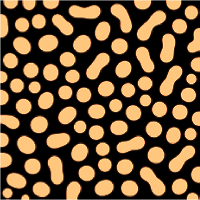

Supplement: S1 File — This file contains patch pattern maps that were obtained from the implementation of the RDE model along three experiments. These maps represent initial stages and final states when patterns reach equilibrium (ZIP) [file pone.0174942.s003.zip › S1 file_patch pattern maps/Patch pattern maps/experiment2/Rin=0.8_R=0.7eq.png]

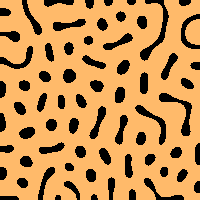

Supplement: S1 File — This file contains patch pattern maps that were obtained from the implementation of the RDE model along three experiments. These maps represent initial stages and final states when patterns reach equilibrium (ZIP) [file pone.0174942.s003.zip › S1 file_patch pattern maps/Patch pattern maps/experiment2/Rin=1.2R=1.1eq.png]

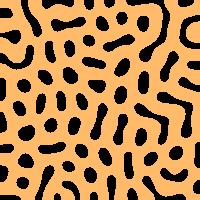

Supplement: S1 File — This file contains patch pattern maps that were obtained from the implementation of the RDE model along three experiments. These maps represent initial stages and final states when patterns reach equilibrium (ZIP) [file pone.0174942.s003.zip › S1 file_patch pattern maps/Patch pattern maps/experiment2/Rin=1.2R=1eq.png]

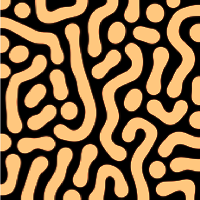

Supplement: S1 File — This file contains patch pattern maps that were obtained from the implementation of the RDE model along three experiments. These maps represent initial stages and final states when patterns reach equilibrium (ZIP) [file pone.0174942.s003.zip › S1 file_patch pattern maps/Patch pattern maps/experiment2/Rin=1_R=0.8eq.png]

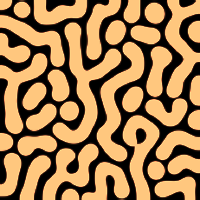

Supplement: S1 File — This file contains patch pattern maps that were obtained from the implementation of the RDE model along three experiments. These maps represent initial stages and final states when patterns reach equilibrium (ZIP) [file pone.0174942.s003.zip › S1 file_patch pattern maps/Patch pattern maps/experiment2/Rin=1_R=0.9eq.png]

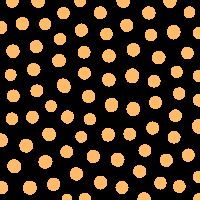

Supplement: S1 File — This file contains patch pattern maps that were obtained from the implementation of the RDE model along three experiments. These maps represent initial stages and final states when patterns reach equilibrium (ZIP) [file pone.0174942.s003.zip › S1 file_patch pattern maps/Patch pattern maps/experiment3/Rin=1.2R=0.6eq.png]

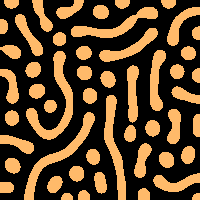

Supplement: S1 File — This file contains patch pattern maps that were obtained from the implementation of the RDE model along three experiments. These maps represent initial stages and final states when patterns reach equilibrium (ZIP) [file pone.0174942.s003.zip › S1 file_patch pattern maps/Patch pattern maps/experiment3/Rin=1.2R=0.7eq.png]

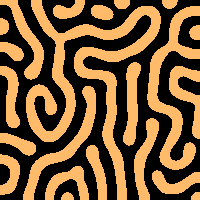

Supplement: S1 File — This file contains patch pattern maps that were obtained from the implementation of the RDE model along three experiments. These maps represent initial stages and final states when patterns reach equilibrium (ZIP) [file pone.0174942.s003.zip › S1 file_patch pattern maps/Patch pattern maps/experiment3/Rin=1.2R=0.8eq.png]

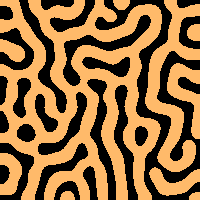

Supplement: S1 File — This file contains patch pattern maps that were obtained from the implementation of the RDE model along three experiments. These maps represent initial stages and final states when patterns reach equilibrium (ZIP) [file pone.0174942.s003.zip › S1 file_patch pattern maps/Patch pattern maps/experiment3/Rin1.2R=0.9eq.png]
